# Genetic and phenotypic stability of *Lacticaseibacillus paracasei* DG (DSM 34154) over 10 years of industrial production

**DOI:** 10.1128/aem.02394-24

**Published:** 2025-04-24

**Authors:** Laura Brunelli, Susanna Perotti, Giorgio Gargari, Valerio De Vitis, Giacomo Mantegazza, Roberto Ferrari, Mario Minuzzo, Elena Pierallini, Giovanni Ricci, Walter Fiore, Simone Guglielmetti

**Affiliations:** 1 Alfasigma S.p.A. 728824 , Milan, Italy; 2 Department of Food, Environmental and Nutritional Sciences (DeFENS), University of Milan 154862, Milan, Italy; 3 μbEat lab, Department of Biotechnology and Biosciences, University of Milano-Bicocca 204547, Milan, Italy; Anses, Maisons-Alfort Laboratory for Food Safety, Maisons-Alfort, France

**Keywords:** probiotic, genetic drift, complete genome, PacBio, INFOGEST, Caco-2, *L. casei *DG

## Abstract

The commercialization of a probiotic microorganism requires genetic and phenotypic consistency across production lots. However, the large-scale industrial production of probiotic microbial biomasses over years poses a risk of genetic drift, potentially affecting the probiotic’s ability to confer health benefits. This study assessed the stability of *Lacticaseibacillus paracasei* DG (DSM 34154), a commercial probiotic. Seven isolates from different commercial lots over 10 years, one 8-year-old laboratory subculture, one isolate from human feces, and the DG strain deposited at DSMZ were analyzed. Shotgun and PacBio sequencing were combined to obtain the complete genome of the 10 isolates. Comparative analysis showed that the seven commercial isolates had identical genomes, differing from the DSMZ isolate by one synonymous transition and one non-synonymous transversion. The laboratory subculture strain had two additional mutations. Phenotypic analyses, including antibiotic resistance, carbohydrate fermentation profile, survival to simulated gastrointestinal transit, immunomodulatory capacity, and radical scavenging ability, found no significant differences among isolates. Overall, this study demonstrates the substantial equivalence of *L. paracasei* DG over 10 years of industrial production, indicating that the current industrial practices help prevent genome alterations that could compromise probiotic performance. Similar studies should be part of continuous monitoring and quality control measures for the probiotic products on the market.

**IMPORTANCE:**

The genetic and functional stability of probiotic strains during years of industrial production is essential but has not been clearly demonstrated for many strains. This study shows that careful industrial practices can maintain the genetic integrity and functionality of probiotics. Using advanced genome sequencing and detailed laboratory tests, we confirmed that the probiotic *Lacticaseibacillus paracasei* DG (DSM 34154) has remained stable over a decade of production, consistently delivering its health-promoting properties. These findings support the quality and reliability of probiotic products, fostering consumer trust and highlighting the importance of continuous monitoring in probiotic manufacturing to sustain quality assurance.

## INTRODUCTION

The market of probiotic products includes a high number of microbial strains, some of which have been commercialized for decades, such as *Lacticaseibacillus rhamnosus* GG, *Bifidobacterium animalis* subsp. *lactis* BB-12, and *Lacticaseibacillus paracasei* Shirota. The latter, considered the first probiotic in history, has been produced and used for over 80 years (1). Various specific capabilities are attributed to these historical probiotic strains, derived from hundreds of scientific studies conducted *in vitro* and in animal models, as well as in clinical trials (1–4). The overall assessment of such historical probiotic strains, based on the results of multiple studies, is founded on the assumption that they have remained unchanged during the uninterrupted multi-decade productions on an industrial scale. However, bacterial genomes are known for their rapid diversification within a short time frame. Following an evolutionary pattern characterized by “quantum leaps” (5), bacterial genome diversification is primarily driven by mobile DNA elements, horizontal gene transfer, genome rearrangement, and related mechanisms (6, 7). Single-nucleotide mutations, occurring randomly, also contribute to a stochastic process known as genetic drift. In contrast to natural selection, which is influenced by specific environmental pressures favoring certain traits, genetic drift involves random changes in the frequencies of gene variants within a bacterial population over time due to chance events (8). Across generations, genetic drift can induce fluctuations in gene variant frequencies, leading to the fixation of a particular gene variant (i.e., it becomes the sole variant in the population), causing other gene variants to disappear (9).

The genetic modifications that may occur in bacterial cells can impact the organism’s performance, potentially altering its ability to provide benefits to the host. Therefore, to mitigate this risk, microbial biomass producers employ strategies to minimize the number of generations from the mother seed in their industrial practices through strict process control during scale up and final fermentation (10, 11). The issue of genetic instability has gained increasing attention also in the context of probiotics following the probable identification of different genotypes of the same strain in probiotic cultures (12, 13). However, there is still a dearth of experimental data that can elucidate whether and to what extent genetic drift events may impact the stability of functional activities in probiotics over the numerous cycles of industrial productions.

In this context, our study aimed to assess the genetic and phenotypic stability of a probiotic bacterium (*Lacticaseibacillus paracasei* DG, DSM 34154) that has been commercially available for more than 20 years through one of the most well-known lines of Italian probiotics (Enterolactis). To achieve this, we obtained and analyzed the complete genome of several isolates of the strain DG, obtained from different commercial lots of Enterolactis produced over approximately 10 years. Furthermore, *in vitro* functional characterization was conducted on the same isolates, analyzing some of the most relevant phenotypes for the probiotic properties of this bacterium, including the ability to survive gastrointestinal transit and the immunomodulatory activity.

## MATERIALS AND METHODS

### Strains under study

**Fig 1:**
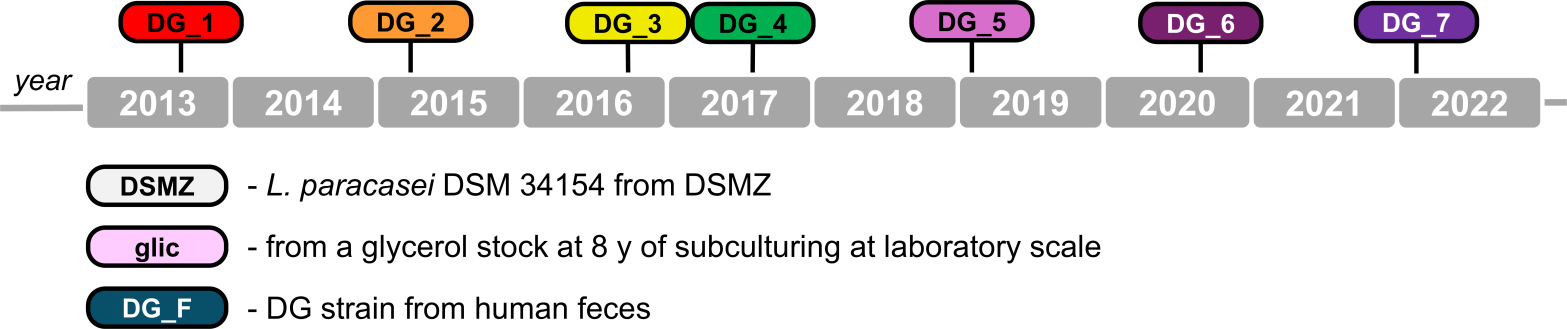
Isolates of *Lacticaseibacillus paracasei* DG used in this study and temporal scale indicating when their respective industrial biomass was produced (DG_1–7) or when the isolation occurred (DG_F).

In this study, 10 isolates of the *Lacticaseibacillus paracasei* DG (L. casei DG) strain were analyzed (Fig. 1). Specifically, we studied the *L. paracasei* DG deposited at the DSMZ culture collection (DSM 34154) (DG_DSMZ) and the DG strain isolated from seven different batches of the commercial probiotic product Enterolactis, containing DG biomasses produced industrially by Christian-Hansen from 2013 to 2022 (DG_1–7) (Fig. 1). To assess whether small-scale laboratory cultivation conditions without control over the number of generations could promote the occurrence of mutations, an isolate of the DG strain obtained from a laboratory-preserved glycerol stock and derived from numerous subcultures over about 8 years was also included in the study (DG_glic). Finally, to investigate whether gastrointestinal transit could induce genetic changes, an isolate of the DG strain was obtained from a fecal sample of a healthy adult who had consumed the Enterolactis product (DG_F; ethic committee approval by the Research Ethics Committee of the Università degli Studi di Milano, opinion no. 37/16, 15th December 2016). The DG_DSMZ was provided in lyophilized form by DSMZ and was cultured in de Man-Rogosa-Sharpe (MRS) broth (Difco Laboratories Inc., Detroit, MI, United States). The seven isolates from Enterolactis products were reactivated in Maximum Recovery Diluent (MRD, Scharlab Italia S.r.l., Riozzo di Cerro al Lambro, Italy) and subsequently cultured in MRS broth. The DG_glic isolate was obtained by inoculating an aliquot of a glycerol stock in 5 mL of MRS broth. Finally, to obtain the DG_F isolate, a fecal sample was serially diluted and plated on MRS agar plates containing kanamycin (10 µg/mL) and vancomycin (1 µg/mL) following the method described by Arioli et al. (14). The identity of each isolate was tested via PCR with the strain-specific primer sets rtWELFf-rtWELFr, targeting the exopolysaccharide-coding region (15) and 8F-8R1, targeting the DG’s major plasmid (16).

### DNA extraction by spooling method

The cell biomass for DNA extraction was obtained culturing *L. paracasei* DG isolates in ten ml of 90% (wt/wt) ISO-Sensitest Broth (Oxoid, Fisher Scientific Italia, Rodano, Italy) and 10% (wt/wt) deMan Rogosa Sharpe (MRS; Difco Laboratories Inc., Detroit, MI, USA) (ISO-MRS broth). The following day, a 0.1% inoculum was made in 50 mL of ISO-MRS broth and incubated overnight at 37°C in anaerobic conditions. Afterward, cells were collected through centrifugation and washed first with 10 mL of a solution containing SDS 0.5%-EDTA 50 mM and then with 10 mL of sterile distilled water. The use of ISO-MRS and thorough biomass washing steps were intended to reduce and remove the exopolysaccharides (EPS) produced by the bacterium before DNA extraction. The pellet was resuspended in 2.5 mL of reagent 1 (containing 125 µL of Tris-HCl 1 M pH 8, 5 µL of EDTA 0.5 M pH 8, and 0.17 g of sucrose) and incubated at 37°C for 5 min in a thermostat-controlled water bath. Subsequently, 625 µL of reagent 2 (containing 15 µL of Tris-HCL 1 M pH 8, and 30 mg of lysozyme) was added, and the mixture was incubated at 37°C for 30 min. Then, 100 µL of 1 mg/mL RNase solution was added, followed by incubation at 37°C for 30 min. At the end of the incubation, 300 µL of reagent 3 (containing 15 µL of Tris-HCl 1M, and 150 µL of EDTA 0.5 M, pH 8) was added and vortexed; then, 200 µL of 20% SDS was added. The resulting suspension was then incubated at 37°C for 10 min. Subsequently, 475 µL of 5 M NaCl was added to the suspension in centrifuge tubes that were then immediately hand-shaken for 2 min. The contents of the centrifuge tubes were transferred to 15 mL Corex glass tubes, and 4 mL of chloroform-isoamyl alcohol (24:1) was added. Then, the samples were incubated overnight at 4°C in the dark. The next day, the Corex tubes were centrifuged at 10,000 rcf for 20 min at 8°C. The upper phase was extracted and transferred into a clean 15 mL Corex tube, adding an equal volume of chloroform-isoamyl alcohol (24:1), and centrifuged again at 10,000 rcf for 20 min at 8°C. The upper phase was carefully extracted and transferred to a 25 mL cylinder to which 2 volumes of −20°C-cold ethanol were added. The DNA was collected on a glass rod by quickly shaking it from top to bottom of the tube. The glass rod was left to dry for 2 h in a laminar flow cabinet, and the DNA was resuspended in 200 µL of 1 × TE buffer (containing 10 mM Tris-HCl, and 1 mM EDTA, pH 8).

### Whole-genome determination and SNP detection analysis

To achieve a high-quality and complete genome assembly of *L. paracasei* DG isolates, we employed a hybrid sequencing approach that combined second-generation (Illumina NovaSeq 6000) and third-generation (PacBio Single Molecule Real-Time, SMRT) sequencing technologies. This strategy leveraged the strengths of both methods: high-throughput short reads from Illumina (2 × 150 bp paired-end) provided a high sequencing depth and accuracy for variant detection and error correction, while long reads from PacBio (>50 × coverage) facilitated the resolution of complex genomic regions, including repetitive sequences and structural variations. Genomic DNA from each isolate was extracted and purified as described above and then sequenced at CD Genomics (Shirley, NY, USA). For shotgun sequencing, we used an Illumina NovaSeq 6000 platform with paired-end and shotgun libraries. After trimming and filtering, we obtained a total of 44,268,368 high-quality paired-end reads (Phred score > 30), with an average of 2,213,418 reads per sample (median: 1,688,979; standard deviation: 1,162,179). These reads were assembled using SPAdes v.3.15.4 (17). PacBio SMRT sequencing was performed to generate long reads with >50 × coverage. To enhance the accuracy of the long-read data, we corrected raw PacBio reads using LoRDEC, which aligns high-accuracy short Illumina reads to error-prone PacBio sequences, thereby reducing sequencing errors (18). The corrected PacBio reads were then assembled *de novo* using Flye v2.9.1, a specialized assembler optimized for long-read sequencing (19). The quality and completeness of the final genome assemblies were assessed using QUAST (Quality Assessment Tool for Genome Assemblies) (20).

To identify single-nucleotide polymorphisms (SNPs) among *L. paracasei* DG genomes, we applied two different variant-calling pipelines. The first approach utilized a suite of bioinformatics tools, including bwa, samtools, and bcftools, with bcftools mpileup as the primary variant-calling command (20). The second method employed Snippy, a specialized tool for bacterial variant calling and core genome alignment from next-generation sequencing (NGS) reads. Both pipelines produced VCF files listing all SNPs detected in each isolate compared to the reference strain DG_DSMZ. Genes containing SNPs were annotated using BLAST, and all identified mutations were further validated by PCR amplification and Sanger sequencing of the surrounding DNA regions.

This integrated sequencing approach ensured the generation of a complete, high-quality genome assembly with minimal sequencing artifacts, providing a robust framework for accurate SNP detection and comparative genomic analyses among isolates.

### Prediction of the functional impact of amino acid substitutions

The potential impact of non-synonymous single-nucleotide polymorphisms on protein function was assessed *in silico* using the Protein Variation Effect Analyzer (PROVEAN) software tool (http://provean.jcvi.org/index.php) (21). The analysis was conducted with default parameters, applying a threshold of −2.5 to classify amino acid variants as either deleterious (score ≤−2.5) or neutral (score >−2.5).

### Antibiotic resistance profiles

*L. paracasei* DG isolates were tested for their sensitivity to eight antibiotics with the method suggested by the European Food Safety Authority (EFSA) (22). In specific, minimum inhibitory concentrations (MICs) were assessed using the broth microdilution method in accordance with the ISO 10932/IDF223:210 protocol. After a pre-inoculation in MRS broth, the 10 isolates of *L. paracasei* DG were inoculated into 10 mL of ISO-MRS and incubated overnight at 37°C under anaerobic conditions. Then, the bacterial suspensions were dispensed in commercial 96-well microtiter plates. The antibiotics tested included ampicillin (0.03–16 µg/mL), gentamicin (0.5–256 µg/mL), kanamycin (2–1,024 µg/mL), chloramphenicol (0.125–64 µg/mL), erythromycin (0.01–8 µg/mL), tetracycline (0.125–64 µg/mL), clindamycin (0.03–16 µg/mL), and streptomycin (0.5–256 µg/mL). For each isolate, a positive control (i.e., inoculated medium without antibiotic) and a negative control (i.e., medium without inoculum) were placed within each plate. Bacterial cells were quantified by flow cytometry and inoculated at a concentration of 10^5^ AFU/ml. *Lacticaseibacillus paracasei* LMG12586 was used as the reference strain according to ISO10932. Finally, turbidity was assessed at O.D._600 nm_ after 48 h of incubation at 37°C under anaerobic conditions. All isolates were tested in triplicate. Additionally, the presence of putative antibiotic resistance genes within the genome of *L. paracasei* DG was investigated using the bioinformatic tool Resistance Gene Identifier (RGI) available on the Comprehensive Antibiotic Resistance Database (CARD) (23).

### Carbohydrate metabolization profiles

The carbohydrate fermentation abilities of the 10 isolates of *L. paracasei* DG were assessed using the API 50 CHL kit (bioMerieux, Durham, NC, USA), following the manufacturer’s instructions. In brief, once centrifuged, the microbial cells in liquid culture were resuspended in Suspension Medium (SM), provided by the kit, to obtain a cell density of 2 in the McFarland Standard scale of turbidity. Approximately 200 µL of this suspension was inoculated into 10 mL of CHL medium, with bromocresol purple used as a pH indicator. Finally, 150 µL of inoculated medium was placed inside each well of the API strip and covered with paraffin. Then, strips were incubated at 37°C. Readings of the strips were taken at both 24 and 48 h. A positive result was indicated by the medium turning yellow due to acidification, as detected by the pH indicator. In the case of esculin metabolization, a black color was observed. A shift from purple to green indicated weak acidification.

This kit allowed the assessment of the following 49 substrates: GLY, glycerol; ERY, erythritol; DARA, D-arabinose; LARA, L-arabinose; RIB, D-ribose; DXYL, D-xylose; LXYL, L-xylose; ADO, D-adonitol; MDX, methyl-beta-D-xylopyranoside; GAL, D-galactose; GLU, D-glucose; FRU, D-fructose; MNE, D-mannose; SBE, L-sorbose; RHA, L-rhamnose; DUL, Dulcitol; INO, inositol; MAN, D-mannitol; SOR, D-sorbitol; MDM, methyl-alpha-D-mannopyranoside; MDG, methyl-α-D-glucopyranoside; NAG, N-acetylglucosamine; AMY, amygdalin; ARB, arbutin; ESC, esculin ferric citrate; SAL, salicin; CEL, D-cellobiose; MAL, D-maltose; LAC, D-lactose (bovine origin); MEL, D-melibiose; SAC, sucrose; TRE, D-trehalose; INU, inulin; MLZ, D-melezitose; RAF, D-raffinose; AMD, starch; GLYG, glycogen; XLT, xylitol; GEN, gentiobiose; TUR, D-turanose; LYX, D-lyxose; TAG, D-tagatose; DFUC, D-fucose; LFUC, L-fucose; DARL, D-arabitol; LARL, L-arabitol; GNT, potassium gluconate; 2KG, potassium 2-ketogluconate; 5KG, potassium 5-ketogluconate.

### Survival to simulated gastrointestinal transit

The resistance of the 10 isolates of *L. paracasei* DG to the gastrointestinal tract was assessed using the INFOGEST 2.0 protocol for *in vitro* static simulation of gastrointestinal digestion (24) as previously described (25). Briefly, after overnight cultivation in MRS broth, 3 × 10⁹ active fluorescent units (AFUs), as determined via flow cytometry with propidium iodide and SYTO24 labeling, were added to 3 mL of simulated salivary fluid. The composition of the simulated fluids and enzymatic activities were standardized according to (24). The digestion process proceeded through three main phases:

- Oral phase: The bacterial suspension was incubated with simulated salivary fluid containing α-amylase (75 U/mL) at 37°C for 2 min under continuous shaking.
- Gastric phase: Simulated gastric fluid (pH adjusted to 3.0 with 5 M HCl) was added along with porcine pepsin (2,000 U/mL) and rabbit gastric extract (60 U/mL lipase). The mixture was incubated at 37°C for 2 h under shaking.
- Intestinal phase: Simulated intestinal fluid (pH adjusted to 7.0 with 5 M NaOH) was introduced, along with porcine pancreatin (providing 100 U/mL trypsin activity) and bovine bile salts (10 mM). The sample was further incubated at 37°C for 2 h.

After the completion of digestion, the final volume was adjusted to 30 mL using maximum recovery diluent (MRD), and serial dilutions were performed in MRS broth supplemented with kanamycin (10 µg/mL) and vancomycin (1 µg/mL). All samples were plated on MRS agar and incubated anaerobically at 37°C for 48 h. All isolates were tested non-aseptically and in triplicate. *L. paracasei* Shirota and *S. thermophilus* mim078 were used as reference control strains.

### Immunomodulatory activity

The immunomodulatory activity was studied through the activation of the nuclear factor κB (NF-κB) using a stable transfected recombinant Caco-2 cell line with the pNiFty2-Seap vector (InvivoGen, Labogen, Rho, Italy), as described by Brunelli et al. (26). In summary, recombinant Caco-2 monolayers at a concentration of 2 × 10^5^ cells/well were cultured for 15 days in the presence of 50 µg/mL zeocin. Subsequently, the monolayers were washed with PBS and incubated with bacterial cells from 10 DG isolates suspended in fresh DMEM containing 100 mM HEPES (pH 7.4), with a multiplicity of infection (MOI) of approximately 100. Pro-inflammatory stimulation of Caco-2 cells was conducted by adding 2 ng/mL interleukin (IL)-1β. After incubation at 37°C for 6 h, the activity of secreted embryonic alkaline phosphatase (SEAP) reporter enzyme was quantified in the supernatant using the QuantiBlue reagent (Invivogen) following the manufacturer’s protocol with a microplate reader (Multiskan SkyHigh, Thermo Fisher Scientific, Waltham, MA) at O.D._620 nm_. Pure SEAP protein directly added to wells in the presence of different treatments was used as a control. Three independent experiments were conducted in technical duplicate for each condition, except for IL-1β (*n* = 6).

### Radical scavenging activity

The 2,2-diphenyl-1-picrylhydrazyl (DPPH) radical scavenging assay was used to assess the free radical scavenging activity of the 10 isolates of *L. paracasei* DG. This activity was measured by combining 500 µL of each bacterial cell concentration (1.0 × 10^10^, 5.0 × 10^9^, 2.5 × 10^9^, 1.0 × 10^9^ cells/mL) with 500 µL of 0.4 mM DPPH-ethanol solution. The control group included 0.1 M phosphate buffer and DPPH-ethanol solution, while the blank group contained suspensions at different concentrations and ethanol. *N*-acetyl-L-cysteine (NAC) was used as the positive control. All mixtures were incubated at 37°C in the dark for 30 min. Samples were centrifuged at 10,000 rcf for 3 min, and the optical absorbance of the supernatant was measured at 517 nm using a Multiskan GO microplate reader (Thermo Fisher Scientific, Waltham, MA). The DPPH radical scavenging ability was calculated using the following equation:


$$Scavenging\;activity\left(\%\right)=\left[1-\left(A_{s}-A_{b}\right)/A_{c}\right]\times 100\%,$$

where *A*_s_, *A*_b_, and *A*_c_ represent the optical absorbance at 517 nm of the sample, blank, and control, respectively. All isolates were analyzed in triplicate.

### Statistical analysis and software

Statistical calculations were performed using the software program GraphPad Prism 8. A Mann-Whitney test was used to ﬁnd the significant differences in data of the experiments for assessing survival to simulated gastrointestinal transit and immunomodulatory activity. Two-way analysis of variance (ANOVA) was performed for data from the experiment of radical scavenging activity. *P* value < 0.05 was considered for statistical significance.

## RESULTS

### Analysis of the genome of the DG isolates

**Fig 2:**
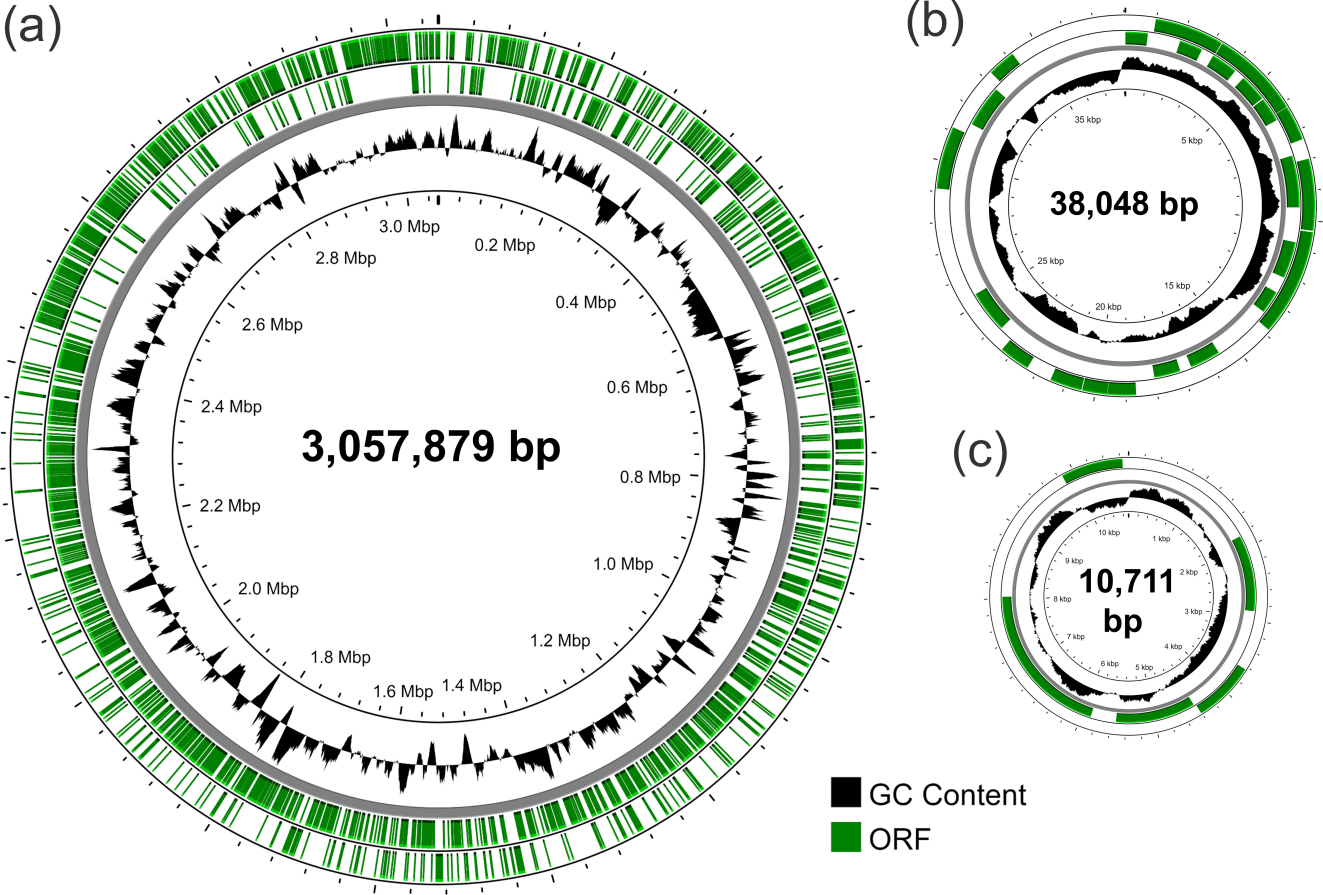
Circular genome maps illustrating the chromosome (**a**), plasmid pDG-L (**b**), and plasmid pDG-S (**c**) of the probiotic strain *Lacticaseibacillus paracasei* DSM 34154 (L. casei DG).

Shotgun and PacBio sequencing data were combined to obtain the complete genome of *Lacticaseibacillus paracasei* DG isolates. For isolate DG_7, however, only a draft genome was obtained since only shotgun data were available. The obtained results revealed that *L. paracasei* DG is a bacterial strain with a genome consisting of a 3,057,879 bp chromosome and two plasmids measuring 38,048 bp (pDG-L) and 10,711 bp (pDG-S), respectively (Fig. 2). Comparative genomic analysis revealed that the only mutations among the isolates’ genomes were nucleotide substitutions, with a total of five different substitutions compared to the reference isolate DG_DSMZ (three transitions and two transversions; Table 1; Fig. S1). All mutations were observed in the chromosome, while the DNA sequences of the two plasmids were identical among isolates. Notably, six out of nine isolates shared an identical complete genome (i.e., DG_2, DG_3, DG_4, DG_5, DG_6, and F).

**TABLE 1:** General genome features of the *Lacticaseibacillus paracasei* DG isolates^*c*^

Isolate	Chromosome size (bp)	Large plasmid pDG-L size (bp)	Small plasmid pDG-S size (bp)	GC content (%)	Alignment against DSM 34154 (%)	No. of point mutations^*a*^
DG_DSMZ	3,057,879	38,048	10,711	46.4	100.000000	Reference
DG_1	3,057,879	38,048	10,711	46.4	99.999934	3
DG_2	3,057,879	38,048	10,711	46.4	99.999902	2
DG_3	3,057,879	38,048	10,711	46.4	99.999934	2
DG_4	3,057,879	38,048	10,711	46.4	99.999934	2
DG_5	3,057,879	38,048	10,711	46.4	99.999934	2
DG_6	3,057,879	38,048	10,711	46.4	99.999934	2
DG_7^*b*^	NA	NA	NA	NA	NA	NA
glic	3,057,879	38,048	10,711	46.4	99.999869	4
F	3,057,879	38,048	10,711	46.4	99.999934	2

^*a*^ Number of mutations compared to DSM 34154 (DG_DSMZ).

^*b*^ Only the draft genome obtained by shotgun sequencing is available.

^*c*^ The complete genome has been determined by combining shotgun and PacBio sequencing data for all isolates except for DG_7, for which only shotgun sequencing data were available. NA, data not available.

Among the five observed mutations, four putatively lead to amino acid substitutions in the respective putative gene, whereas one is in a putative non-coding region (Table 2). In specific, two mutations affected exclusively the DG isolate “glic”: (i) the mutation in the non-coding region and (ii) a G > T transition resulting in Asn > Lys substitution in a putative 123 amino acid-long protein sharing significant homology with the large-conductance mechanosensitive channel protein MscL, which was shown in *Escherichia coli* to be involved in the transduction of membrane osmotic stress (mechanical or osmotic) into an electrochemical response (for references, see GenBank gene ID: 947787). Other two mutations were present in all isolates compared to DG_DSMZ: (i) A > G silent transition (Asp/Asp) in a putative 554 amino acid-long dihydrolipoyllysine-residue acetyltransferase (E2) component of the pyruvate dehydrogenase complex that catalyzes the conversion of pyruvate to acetyl-CoA (accession code PRK11855 in the NCBI conserved domain database) and (ii) C > G transversion resulting into Pro > Arg substitution in a putative 635 amino acid-long protein with a significant similarity with the NADH dehydrogenase (Ndh)-like protein YjlD (GenBank gene ID: 11639487). However, this mutation was found in a region outside the putative catalytic domain (Fig. S1). Finally, we found in DG_1 a T > C transition resulting into Ile > Val substitution in a putative alpha subunit of the FoF1-type ATP synthase (Table 2; Fig. S1).

**TABLE 2:** Summary of the mutations observed in the complete *Lacticaseibacillus paracasei* DG genomes^*c*^

Mutation	Position^*a*^	DSM 34154	*L. paracasei* DG isolates									Putative function	Amino acid replacement	PROVEAN results		
			1	2	3	4	5	6	7^*b*^	glic	F			Variant	Score	Prediction
G → T	1304422	G	G	G	G	G	G	G	G	T	G	Large-conductance mechanosensitive channel protein MscL	Asn > Lys	N37K	−5.060	Deleterious
C → G	1567535	C	G	G	G	G	G	G	G	G	G	NADH dehydrogenase-like protein YjlD	Arg > Pro	R430P	8.145	Neutral
G → A	2198112	G	G	G	G	G	G	G	G	A	G	Putative noncoding region	/	/	/	/
A → G	2574335	A	G	G	G	G	G	G	G	G	G	Dihydrolipoamide acetyltransferase	Asp > Asp	/	/	/
T → C	2710129	T	C	T	T	T	T	T	T	T	T	FoF1-type ATP synthase, alpha subunit	Ile > Val	I451V	−0.420	Neutral

^*a*^ Positions on the chromosome are according to the map of Fig. 2.

^*b*^ Only shotgun sequencing data are available.

^*c*^ The nucleotide substitutions compared to the reference sequence (DSM 34154) are highlighted with a gray background. Asp, aspartic acid; Arg, arginine; Lys, lysine; Pro, proline. The final three columns present the results of the Protein Variation Effect Analyzer (PROVEAN) analysis, which assesses the potential impact of amino acid substitutions on the biological function of the predicted proteins. The PROVEAN score was computed using the default threshold of −2.5: variants with a score ≤ −2.5 are classified as "deleterious," while those with a score > −2.5 are considered "neutral." /, not applicable (the affected region is putatively not protein-coding).

To assess the potential impact of identified non-synonymous mutations on protein function, we employed the PROVEAN software tool. This analysis classified only the N37K substitution in the putative Mscl protein of DG_glic as deleterious (PROVEAN score: −5.060), suggesting a potential functional impairment. In contrast, the R430P substitution in the NADH dehydrogenase-like protein was predicted to be neutral (score: 8.145), indicating no significant effect on protein function. Similarly, the I451V mutation in the FoF1-type ATP synthase α-subunit was also classified as neutral (score: −0.420). The remaining two substitutions were not analyzed, as one resulted in a synonymous change (Asp > Asp), while the other was located in a putative non-coding region (Table 2).

### Phenotypic and functional characterization of the DG isolates

The 10 isolates of *L. paracasei* DG were subjected to functional characterization through five assays conventionally used for the study of probiotic microbial strains. The obtained results are described below.

#### Antibiotic resistance profiles

The results of the analysis of the antibiotic resistance profile, investigated according to ISO 10932 IDF223:210, have revealed that there are no significant differences among the ten isolates of *L. paracasei* DG (Table 3). The minimum inhibitory concentrations (MICs) calculated for strain DG fell within the EFSA breakpoints for ampicillin, chloramphenicol, clindamycin, erythromycin, streptomycin, and tetracycline. Compared to EFSA breakpoints, the resistance was significantly higher for gentamicin (7.6 ± 2.0 µg/mL compared to a breakpoint of 1–4 μg/mL) and kanamycin (124 ± 13 µg/mL compared to a breakpoint of 16–64 μg/mL). In addition, the use of the CARD bioinformatic tool revealed the presence of a putative gene coding for a multidrug transporter EmrE; nonetheless, the same gene is common within the members of the *L. paracasei* species, such as the well-known probiotic strains Shirota and CBA L74 (Table S1).

**TABLE 3:** Minimum inhibitory concentrations (MICs) of *Lacticaseibacillus paracasei* DG isolates determined using the microdilution assay described in the ISO 10932 (IDF 223) document and recommended by EFSA (22)^*c*^

EFSA breakpoints^*a*^	Streptomycin	Kanamycin	Ampicillin	Erythromycin	Gentamicin	Chloramph	Clindamycin	Tetracycline
	8–32	16–64	0.5–2	0.062–0.25	1–4	1–4	0.062–0.25	1–4
LMG 12586^*b*^	8; 16; 16	32; 32; 32	1; 0.5; 0.5	0.03; <0.01; <0.01	2; 1; 2	4; 4; 4	<0.03; <0.03; <0.03	1; 2; 0.5
DSM 34154	32; 32; 32	128; 128; 128	2; 2; 1	0.03; 0.06; <0.01	4; 4; 8	8; 4; 4	<0.03; 0.06; <0.03	1; 1; 1
DG_1	32; 32; 32	128; 128; 128	1; 2; 2	0.06; 0.06; <0.01	8; 4; 16	8; 4; 4	0.06; <0.03; <0.03	2; 1; 1
DG_2	32; 32; 32	128; 128; 128	1; 2; 1	0.06; 0.06; <0.01	8; 4; 16	4; 4; 4	<0.03; <0.03; <0.03	1; 1; 1
DG_3	32; 32; 32	128; 128; 128	2; 2; 1	0.06; 0.03; <0.01	8; 8; 8	4; 4; 4	<0.03; 0.06; <0.03	1; 1; 1
DG_4	32; 32; 64	64; 128; 128	1; 2; 2	0.03; 0.03; <0.01	4; 4; 4	4; 4; 4	<0.03; <0.03; <0.03	1; 1; 1
DG_5	32; 32; 32	128; 128; 128	2; 2; 1	0.03; 0.06; <0.01	4; 8; 8	4; 8; 4	<0.03; 0.06; <0.03	1; 1; 0.5
DG_6	32; 64; 32	128; 128; 128	1; 2; 1	0.06; 0.06; <0.01	8; 8; 8	4; 4; 4	0.06; <0.03; <0.03	1; 1; 0.5
DG_7	32; 32; 32	64; 128; 256	2; 2; 1	0.03; 0.03; <0.01	4; 8; 8	4; 8; 4	<0.03; 0.06; <0.03	1; 2; 0.5
DG_F	32; 32; 32	64; 128; 128	1; 1; 1	0.06; 0.06; <0.01	16; 8; 8	8; 4; 4	0.06; <0.03; <0.03	2; 1; 1
DG_glic	32; 32; 32	128; 128; 64	1; 2; 0.5	0.06; 0.06; <0.01	4; 4; 16	4; 4; 4	<0.03; 0.06; <0.03	1; 1; 1

^*a*^ Cut-off values defined by EFSA for distinguishing resistant strains from susceptible strains within the taxonomic group *Lactobacillus casei*/*paracasei*.

^*b*^ Technical reference strain.

^*c*^ Data are reported as μg/mL.

#### Carbohydrate metabolization profiles

According to the analysis conducted with the API 50 CHL kit, all *L. paracasei* DG isolates displayed an identical carbohydrate utilization profile both after 24 and 48 h. In specific, all DG isolates were able to metabolize the following carbohydrates: RIB, ADO, GAL, GLU, FRU, MNE, SBE, MAN, SOR, NAG, ARB, ESC, SAL, CEL, MAL, SAC, TRE, INU, MLZ, TUR, and TAG. A weak positive reaction was also observed for INO, AMY, AMD, GEN, LYX, and GNT. The other 22 substrates present in the kit were not metabolized (Table 4).

**TABLE 4:** Results of the biochemical tests carried out by means of the API 50 CHL system^*a*^

	C	GLY	ERY	DARA	LARA	RIB	DXYL	LXYL	ADO	MDX	GAL	GLU	FRU	MNE	SBE	RHA	DUL	INO	MAN	SOR	MDM	MDG	NAG	AMY	ARB	ESC	SAL	CEL	MAL	LAC	MEL	SAC	TRE	INU	MLZ	RAF	AMD	GLYG	XLT	GEN	TUR	LYX	TAG	DFUC	LFUC	DARL	LARL	GNT	2KG	5KG
DG_DSMZ	−	−	−	−	−	+	−	−	+	−	+	+	+	+	+	−	−	*w*	+	+	−	−	+	*w*	+	+	+	+	+	−	−	+	+	+	+	−	*w*	−	−	*w*	+	*w*	+	−	−	−	−	*w*	−	−
DG_1	−	−	−	−	−	+	−	−	+	−	+	+	+	+	+	−	−	*w*	+	+	−	−	+	*w*	+	+	+	+	+	−	−	+	+	+	+	−	*w*	−	−	*w*	+	*w*	+	−	−	−	−	*w*	−	−
DG_2	−	−	−	−	−	+	−	−	+	−	+	+	+	+	+	−	−	*w*	+	+	−	−	+	*w*	+	+	+	+	+	−	−	+	+	+	+	−	*w*	−	−	*w*	+	*w*	+	−	−	−	−	*w*	−	−
DG_3	−	−	−	−	−	+	−	−	+	−	+	+	+	+	+	−	−	*w*	+	+	−	−	+	*w*	+	+	+	+	+	−	−	+	+	+	+	−	*w*	−	−	*w*	+	*w*	+	−	−	−	−	*w*	−	−
DG_4	−	−	−	−	−	+	−	−	+	−	+	+	+	+	+	−	−	*w*	+	+	−	−	+	*w*	+	+	+	+	+	−	−	+	+	+	+	−	*w*	−	−	*w*	+	*w*	+	−	−	−	−	*w*	−	−
DG_5	−	−	−	−	−	+	−	−	+	−	+	+	+	+	+	−	−	*w*	+	+	−	−	+	*w*	+	+	+	+	+	−	−	+	+	+	+	−	*w*	−	−	*w*	+	*w*	+	−	−	−	−	*w*	−	−
DG_6	−	−	−	−	−	+	−	−	+	−	+	+	+	+	+	−	−	*w*	+	+	−	−	+	*w*	+	+	+	+	+	−	−	+	+	+	+	−	*w*	−	−	*w*	+	*w*	+	−	−	−	−	*w*	−	−
DG_7	−	−	−	−	−	+	−	−	+	−	+	+	+	+	+	−	−	*w*	+	+	−	−	+	*w*	+	+	+	+	+	−	−	+	+	+	+	−	*w*	−	−	*w*	+	*w*	+	−	−	−	−	*w*	−	−
DG_glic	−	−	−	−	−	+	−	−	+	−	+	+	+	+	+	−	−	*w*	+	+	−	−	+	*w*	+	+	+	+	+	−	−	+	+	+	+	−	*w*	−	−	*w*	+	*w*	+	−	−	−	−	*w*	−	−
DG_F	−	−	−	−	−	+	−	−	+	−	+	+	+	+	+	−	−	*w*	+	+	−	−	+	*w*	+	+	+	+	+	−	−	+	+	+	+	−	*w*	−	−	*w*	+	*w*	+	−	−	−	−	*w*	−	−

^*a*^ The gray scale, from lightest to darkest, visually distinguishes the three categories of results: +, positive result in the test; w, weak positivity; −, the substrate is not metabolized. C, negative control, GLY, Glycerol; ERY, Erythritol; DARA, D-arabinose.; LARA, L-arabinose; RIB, D-ribose; DXYL, D-xylose; LXYL, L-xylose; ADO, D-xylose; MDX, Methyl-beta-D-xylopyranoside; GAL, D-galactose; GLU, D-glucose; FRU, D-fructose; MNE, D-mannose; SBE, L-sorbose; RHA, L-rhamnose; DUL, Dulcitol; INO, Inositol; MAN, D-mannitol; SOR, D-sorbitol; MDM, Methyl-alpha-D-mannopyranoside; MDG, Methyl-α**-**D-glucopyranoside; NAG, N-acetylglucosamine; AMY, Amygdalin; ARB, Arbutin; ESC, Esculin ferric citrate; SAL, Salicin; CEL, D-cellobiose; MAL, D-maltose; LAC, D-lactose (bovine origin); MEL, D-melibiose; SAC, Sucrose; TRE, D-trehalose; INU, Inulin; MLZ, D-melezitose; RAF, D-raffinose; AMD, Starch; GLYG, Glycogen; XLT, Xylitol; GEN, Gentiobiose; TUR, D-turanose; LYX, D-lyxose; TAG, D-tagatose; DFUC, D-fucose; LFUC, L-fucose; DARL, D-arabitol; LARL, L-arabitol; GNT, Potassium gluconate; 2KG, Potassium 2-ketogluconate; 5KG, Potassium 5-ketogluconate.

#### Survival of L. paracasei DG during simulated gastrointestinal transit

**Fig 3:**
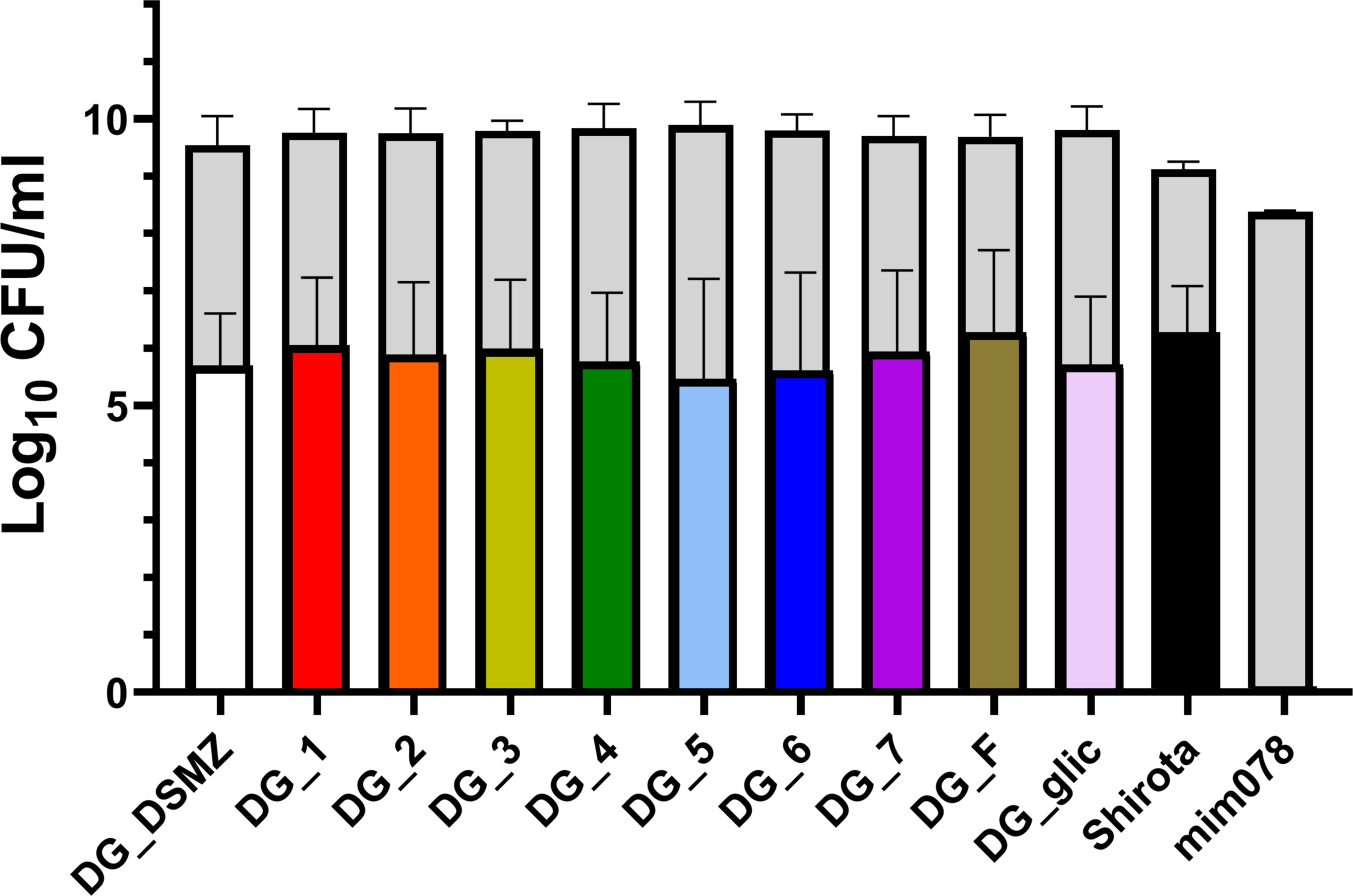
Survival of *Lacticaseibacillus paracasei* DG isolates was evaluated using *in vitro* static simulated gastrointestinal digestion (INFOGEST protocol). *Lacticaseibacillus paracasei* Shirota and *Streptococcus thermophilus* mim078 were utilized as positive and negative controls for survival, respectively. Grey histograms represent the bacterial load before INFOGEST, while white, black, and colored histograms indicate the corresponding bacterial load after INFOGEST. Data for DG isolates are derived from *n* = 6 independent experiments, while data for strains Shirota and mim078 are derived from *n* = 5 and *n* = 2 independent experiments, respectively. The statistical analysis, conducted using the Mann-Whitney test on the variations in microbial load before and after INFOGEST, showed no significant differences among the DG isolates.

We utilized the INFOGEST static *in vitro* simulation of gastrointestinal digestion to assess the capability of the 10 isolates of *L. paracasei* DG under digestive tract conditions. The results showed that the 10 isolates exhibited similar abilities to survive the simulation of gastrointestinal transit. Specifically, starting from a mean of 7.9 × 10^9^ CFU/mL, the 10 isolates had a reduction of approximately 3 logs, reaching a final count of 6.3 × 10^6^ CFU/mL. No significant differences were recorded among the 10 DG isolates. The positive control *Lacticaseibacillus paracasei* Shirota exhibited a slightly lower reduction than DG isolates (−2.6 log), while no survival in the gastrointestinal tract was recorded for the negative control, *S. thermophilus* mim078 (Fig. 3).

#### Immunomodulatory activity

**Fig 4:**
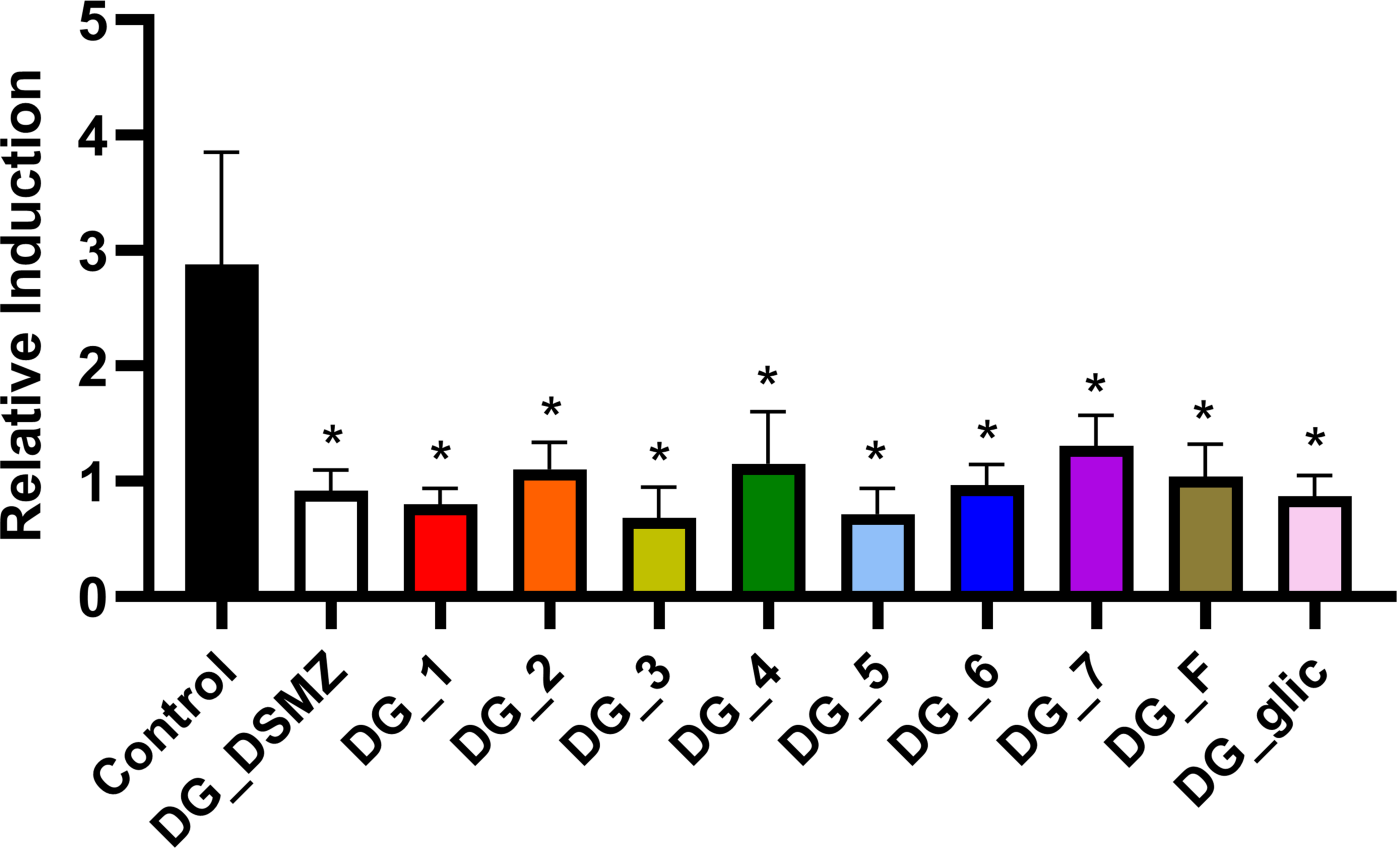
Activation of the nuclear factor κB (NF-κB) in intestinal epithelial cells. The experiment was conducted using a recombinant Caco-2 cell line stably transfected with the pNiFty2-SEAP vector containing an alkaline phosphatase reporter gene. Pro-inflammatory stimulation of the Caco-2 cells was achieved by adding 2 ng/mL of interleukin (IL)-1β. A multiplicity of infection (MOI) of 100 was used. Three independent experiments were performed in technical duplicate for each condition, except for the control (*n* = 6). Asterisks (*) indicate significant differences compared to the control. Statistical analysis was performed using the Mann-Whitney test. *P < 0.05.

The ability of DG isolates to modulate host immune responses was studied by assessing the activation of the transcriptional factor NF-κB in differentiated epithelial Caco-2 cells stably transfected with the pNiFty2-SEAP vector. In specific, DG cells at a MOI of 100 were incubated with recombinant Caco-2 cells in the presence of the pro-inflammatory cytokine IL-1β (2 ng/mL). All DG isolates significantly reduced NF-κB activation in this cell model. Specifically, we observed an average reduction of 66.9 ± 6.9% in NF-κB activation, with no statistically significant differences among isolates (Fig. 4).

#### Radical scavenging activity of *Lacticaseibacillus paracasei* DG

**Fig 5:**
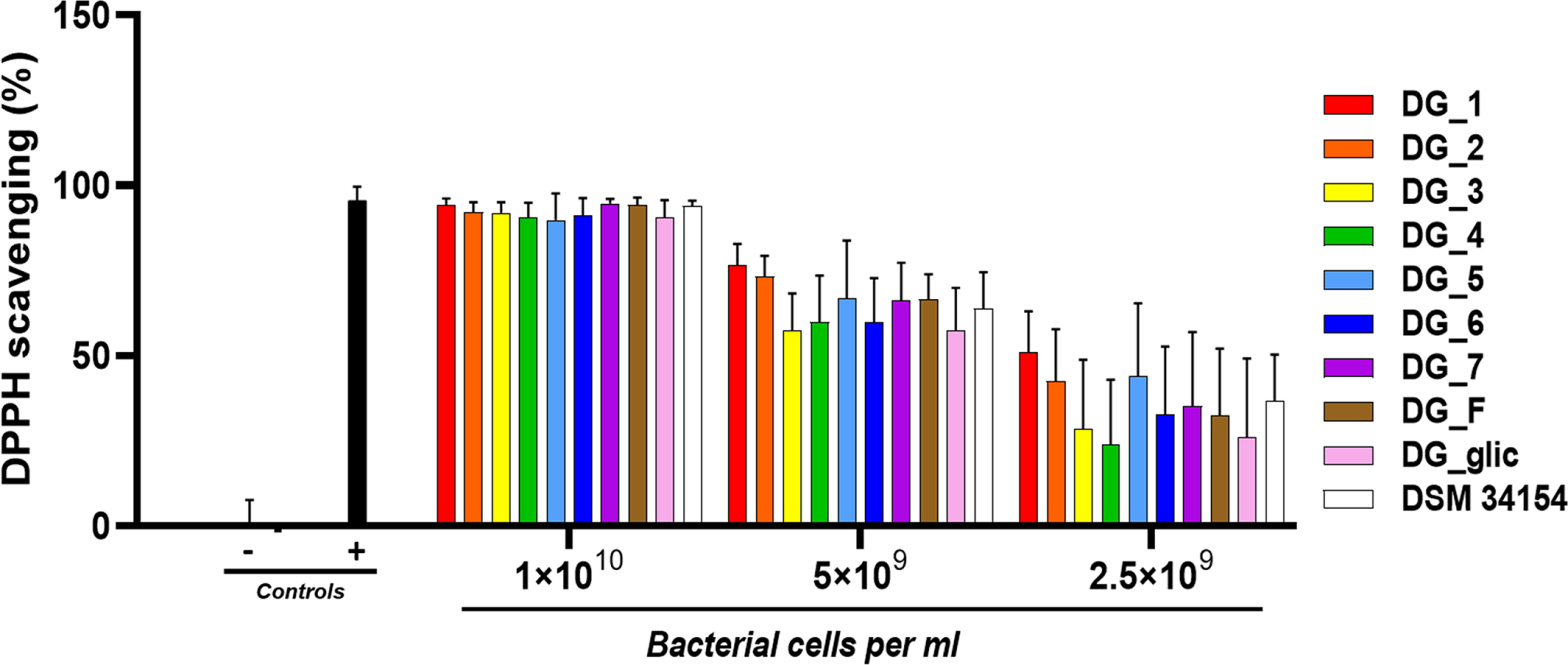
Radical scavenging activity of *Lacticaseibacillus paracasei* DG. The experiment was carried out incubating three different concentrations of DG cells with 0.4 mM 2,2-diphenyl-1-picrylhydrazyl (DPPH) at 25°C in the dark for 30 min. Data reported in the histogram derive from three independent experiments. The negative control (−) consisted of a DPPH solution without DG cells, while the positive control (+) comprised 10 mg/mL *N*-acetyl cysteine. No significant differences were found among isolates according to two-way ANOVA (*P* = 0.753).

We evaluated the antioxidant properties of *L. paracasei* DG isolates by assessing their ability to scavenge DPPH radicals. The results revealed that the highest quantity of bacterial cells tested (10 billion cells) exhibited radical scavenging activity comparable to that of the positive control (10 mg/mL *N*-acetyl cysteine). As the bacterial cell numbers decreased, the radical scavenging activity progressively diminished. However, the DG isolates did not demonstrate significant differences among each other (Fig. 5).

## DISCUSSION

Commercializing a probiotic strain relies on the assumption that microbial cells within production lots are identical. However, continuous industrial productions may lead to DNA mutations, which can progressively accumulate during generations, potentially generating novel genetic variants. Such potential genetic drift in industrial probiotic strains is not considered a safety risk, but it may impact probiotic performance and compromise the intended health benefits. Nonetheless, while historical probiotic strains have been widely used and investigated for decades, the genetic drift in these microbes was largely understudied.

Based on what is known about genetic drift in bacterial populations, two theoretical frameworks can be considered, leading to divergent conclusions for industrial biomasses of probiotic microbes. (i) The first hypothesis starts from the premise that genetic drift is more pronounced in smaller bacterial populations, where chance events can exert a greater influence on genetic makeup, whereas an increase in population size determines a dilution effect that reduces drift. Therefore, it can be argued that genetic drift may have a limited impact on probiotic biomasses, which are produced industrially in thousand liters bioreactors in very large populations of microbial cells. (ii) The second hypothesis is based on the assertion that the rate of nonsynonymous substitution per site is typically lower than the rate of synonymous substitution per site in functional genes. This is because point mutations that lead to amino acid replacements are often deleterious. In the context of industrial cultivation conditions, a higher number of genes, particularly those involved in interactions with the environment (including host and other microorganisms), may become not necessary anymore for survival. Therefore, the condition of limited selective pressures in industrial bioreactors for these genes could theoretically result in an increased overall rate of nonsynonymous substitution and nucleotide deletion per site. This, in turn, may lead to a reduction in gene density through the creation of pseudogenes, potentially involving genes contributing to the manifestation of probiotic properties. Given the very limited research in this area, it is not possible to establish the actual impact of these mutation events on industrial probiotic microorganisms.

The fact that genetic drift could be a concrete issue for probiotics is suggested by only a few studies, the most notable of which is that of Sybesma et al. (12), who reported the presence of genetic variants of the *Lacticaseibacillus rhamnosus* GG strain characterized by the deletion in the *spaCBA-srtC1* gene cluster. This operon encodes the protein subunits that constitute the pili of strain GG, which have been shown to be involved in adhesion and immunomodulation properties of this bacterium (27). Importantly, the *spaCBA-srtC1* gene cluster resides in a region abundant in insertion sequences, which can induce instability. This was also observed in another study where deletions of the *spaCBA-srtC1* gene cluster occurred in some isolates after 1,000 generations under laboratory conditions with continuous exposure to bile or mechanical stress (28). A subsequent study, however, evaluated the genetic stability of this bacterium under industrial production conditions, concluding that *L. rhamnosus* GG is highly stable genetically and phenotypically, considering it is strongly unlikely that the genetic loss observed by Sybesma et al. could occur as a result of the production process of *L. rhamnosus* GG (29). However, the Stage et al. study was conducted over a reduced time scale (i.e., with a reduced number of generations). The same authors reported good genome conservation when the GG strain was co-fermented in yogurt (30).

Besides *L. rhamnosus* GG, the genetic stability during industrial productions was only limitedly assessed in other probiotic strains. Due to a lack of experimental data in this field, we initiated an investigation into the stability of the probiotic strain *Lacticaseibacillus paracasei* DG. In particular, we isolated the bacterium from seven commercial Enterolactis products containing industrial biomasses produced between August 2013 and March 2022. We isolated the bacterium from the final product in order to test what is actually assumed by the end consumer. These isolates were then compared with the same strain obtained from the DSMZ strain collection (DSM 34154) and with an isolate obtained from a glycerol stock of a culture used in the laboratory for several years to assess whether continuous subculturing in small-scale laboratory conditions in MRS medium may have influenced the genetic variation rate. The study then included a fecal isolate to evaluate the stability of the bacterium of interest also *in vivo* in the host.

*Lacticaseibacillus paracasei* DG (DSM 34154) is the bacterial strain used in the Enterolactis line of dietary supplements, which represents one of the most widely sold probiotic products in Italy. Previous studies have demonstrated that *L. paracasei* DG effectively survives gastrointestinal transit in both adults (14) and children (31). In our study, we evaluated this ability *in vitro*, using the standardized protocol of *in vitro* simulation of gastrointestinal digestion [INFOGEST (24)], through which we confirmed the good survival capabilities of the DG strain and did not find any significant difference among the 10 isolates.

Antibiotic resistance is another crucial aspect for probiotics (32), and in this study, it was assessed using the ISO/IDF microdilution assay recommended by EFSA, applying EFSA breakpoints for the identification of potential acquired antibiotic resistance genes (22). We found a resistance to gentamicin and kanamycin modestly higher than the EFSA breakpoints. We previously reported a similar result for kanamycin with strain DG (14). High natural resistance to aminoglycosides, such as gentamicin and kanamycin, has often been observed in lactobacilli (33). This, coupled with the abundant EPS in strain DG (34), suggests that the observed resistance to kanamycin is plausibly an intrinsic (non-transmissible) feature of *L. paracasei* DG. These conclusions are corroborated by the fact we did not find any known putative transmissible antibiotic resistance genes when we analyzed *in silico* the DG genome.

Feng et al. studied the phenotypic stability of a *Lactiplantibacillus plantarum* strain following daily subculture in laboratory medium (10% MRS and 90% ISO-Sensitest broth) over a 90-day period. Notably, API 50 CHL kit analysis revealed a change in carbohydrate metabolism patterns after 30 subcultures (13). In our study, we used the same biochemical assay, yet we observed no differences in substrate utilization characteristics among the 10 DG isolates.

To assess immunomodulatory activity, we tested the ability of DG to reduce NF-κB activation in Caco-2 cells, given its previously demonstrated anti-inflammatory potential in the same *in vitro* model (15, 26). Additionally, we also recently reported the ability of an industrial preparation of two *L. paracasei* strains, including the DG strain, to exert cellular antioxidant activity in Caco-2 cells (26). Building on these findings, we assessed the same properties for DG isolates also in this study. Similar to previous characterization experiments, we did not observe any significant differences among DG isolates in terms of immunomodulation and antioxidant properties, further supporting the functional stability of the probiotic strain *L. paracasei* DG.

A limitation of our study is that we focused on a single isolate from each commercial product, without accounting for the potential heterogeneity in the original industrial biomasses, i.e., the presence of genetic variants. However, given that all isolates were found to be substantially isogenic to the reference DG strain, our data suggest that the strain under study exhibits high genetic stability under the adopted industrial production conditions. If any genetic variants emerged, they did not supplant the original genotype or become predominant. Our findings do not necessarily imply that the original production conditions were equally favorable to all genetic variants that may have been present at the beginning of the process. Rather, they suggest that only those strains capable of enduring these conditions persisted. Nonetheless, our results confirm that the retained strain remains genetically stable and preserves its probiotic properties over time, reinforcing the reliability of current production practices.

### Conclusion

Our findings establish the substantial equivalence of the *L. paracasei* DG strain from industrial productions over the past decade, ensuring the consistency of its probiotic properties. Therefore, our study emphasizes the effectiveness of current industrial practices in producing the DG strain, preventing genome alterations that could compromise the performance of this probiotic bacterium. This assures both producers and consumers of the preservation of the intended health benefits over time of the probiotic products containing this bacterium.

Understanding genetic drift in probiotics is crucial for ensuring product stability, emphasizing the importance of continuous monitoring and quality control measures. We believe there is a need for similar studies on other commercially significant probiotic strains to reinforce probiotic product reliability in the market.

## Data Availability

Sequencing data have been deposited in the European Nucleotide Archive (ENA) of the European Bioinformatics Institute under accession code PRJEB82825. Other data, including those related to the functional characterization of the isolates, analyzed during the current study are available from the corresponding author upon reasonable request.

## SUPPLEMENTAL MATERIAL

Figure S1 aem.02394-24-s0001.pdf ClustalW multiple sequence alignment of the amino acid sequences translated from the three putative genes where non-conservative mutations have been identified.

Table S1 aem.02394-24-s0002.xlsx Output of the bioinformatic tool Resistance Gene Identifier (RGI) available on the Comprehensive Antibiotic Resistance Database (CARD) used with the genome of the probiotic *Lacticaseibacillus paracasei* strains Shirota, CBA L74, and DG.

ASM does not own the copyrights to Supplemental Material that may be linked to, or accessed through, an article. The authors have granted ASM a non-exclusive, world-wide license to publish the Supplemental Material files. Please contact the corresponding author directly for reuse.
