# Supplementary material for: Genetic and phenotypic stability of Lacticaseibacillus paracasei DG (DSM 34154) over 10 years of industrial production
Source: Appl Environ Microbiol. 2025 Apr 24;91(5):e02394-24. doi: 10.1128/aem.02394-24 (PMC12093954; doi:10.1128/aem.02394-24)
Supplement: Figure S1 — ClustalW multiple sequence alignment of the amino acid sequences translated from the three putative genes where non-conservative mutations have been identified. [file aem.02394-24-s0001.pdf]

**Supplementary Figure 1**. ClustalW multiple sequence alignment of the aminoacidic sequence translated from the three putative genes where non-conservative mutations have been identified. (**A**) Large-conductance mechanosensitive channel protein MscL. (**B**) Ndh, NADH dehydrogenase, FAD-containing subunit; DocX, Uncharacterized membrane protein YphA, DoxX/SURF4 family. (**C**) FoF1-type ATP synthase, alpha subunit. The pictures before the alignments derive from the output of the NCBI Conserved Domain Database search (https://www.ncbi.nlm.nih.gov/Structure/cdd/wrpsb.cgi). The vertical red arrows indicate the position of the mutation. ClustalW symbols: "*" means that the residues or nucleotides in that column are identical in all sequences in the alignment; ":" means conserved substitutions.

(**A**)

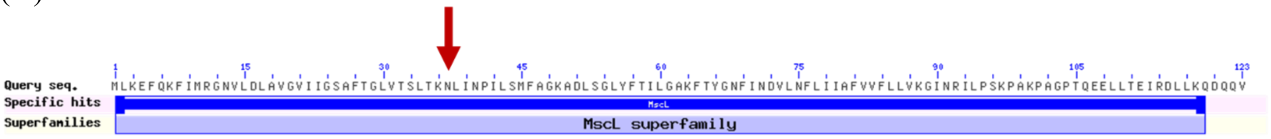


```
DG_DSM          MLKEFQKFIMRGNVLDLAVGVIIGSAFTGLVTSLTKNLINPILSMFAGKADLSGLYFTIL
DG_1            MLKEFQKFIMRGNVLDLAVGVIIGSAFTGLVTSLTKNLINPILSMFAGKADLSGLYFTIL
DG_2            MLKEFQKFIMRGNVLDLAVGVIIGSAFTGLVTSLTKNLINPILSMFAGKADLSGLYFTIL
DG_3            MLKEFQKFIMRGNVLDLAVGVIIGSAFTGLVTSLTKNLINPILSMFAGKADLSGLYFTIL
DG_4            MLKEFQKFIMRGNVLDLAVGVIIGSAFTGLVTSLTKNLINPILSMFAGKADLSGLYFTIL
DG_5            MLKEFQKFIMRGNVLDLAVGVIIGSAFTGLVTSLTKNLINPILSMFAGKADLSGLYFTIL
DG_6            MLKEFQKFIMRGNVLDLAVGVIIGSAFTGLVTSLTKNLINPILSMFAGKADLSGLYFTIL
DG_7            MLKEFQKFIMRGNVLDLAVGVIIGSAFTGLVTSLTKNLINPILSMFAGKADLSGLYFTIL
DG_F            MLKEFQKFIMRGNVLDLAVGVIIGSAFTGLVTSLTKNLINPILSMFAGKADLSGLYFTIL
DG_glic         MLKEFQKFIMRGNVLDLAVGVIIGSAFTGLVTSLTKKLINPILSMFAGKADLSGLYFTIL
                ************************************:***********************

DG_DSM          GAKFTYGNFINDVLNFLIIAFVVFLLVKGINRILPSKPAKPAGPTQEELLTEIRDLLKQD
DG_1            GAKFTYGNFINDVLNFLIIAFVVFLLVKGINRILPSKPAKPAGPTQEELLTEIRDLLKQD
DG_2            GAKFTYGNFINDVLNFLIIAFVVFLLVKGINRILPSKPAKPAGPTQEELLTEIRDLLKQD
DG_3            GAKFTYGNFINDVLNFLIIAFVVFLLVKGINRILPSKPAKPAGPTQEELLTEIRDLLKQD
DG_4            GAKFTYGNFINDVLNFLIIAFVVFLLVKGINRILPSKPAKPAGPTQEELLTEIRDLLKQD
DG_5            GAKFTYGNFINDVLNFLIIAFVVFLLVKGINRILPSKPAKPAGPTQEELLTEIRDLLKQD
DG_6            GAKFTYGNFINDVLNFLIIAFVVFLLVKGINRILPSKPAKPAGPTQEELLTEIRDLLKQD
DG_7            GAKFTYGNFINDVLNFLIIAFVVFLLVKGINRILPSKPAKPAGPTQEELLTEIRDLLKQD
DG_F            GAKFTYGNFINDVLNFLIIAFVVFLLVKGINRILPSKPAKPAGPTQEELLTEIRDLLKQD
DG_glic         GAKFTYGNFINDVLNFLIIAFVVFLLVKGINRILPSKPAKPAGPTQEELLTEIRDLLKQD
                ************************************************************

DG_DSM          QQV
DG_1            QQV
DG_2            QQV
DG_3            QQV
DG_4            QQV
DG_5            QQV
DG_6            QQV
DG_7            QQV
DG_F            QQV
DG_glic         QQV
                ***
```

(**B**)

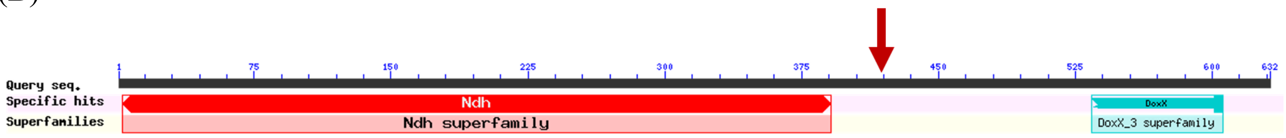


```
DG_glic             MAKTNIVVVGAGFAGVYATKHLAKHYKRNKDVTITLIDRHSYFTYVTELHEIAADRVPED
DG_F                MAKTNIVVVGAGFAGVYATKHLAKHYKRNKDVTITLIDRHSYFTYVTELHEIAADRVPED
DG_7                MAKTNIVVVGAGFAGVYATKHLAKHYKRNKDVTITLIDRHSYFTYVTELHEIAADRVPED
DG_6                MAKTNIVVVGAGFAGVYATKHLAKHYKRNKDVTITLIDRHSYFTYVTELHEIAADRVPED
DG_5                MAKTNIVVVGAGFAGVYATKHLAKHYKRNKDVTITLIDRHSYFTYVTELHEIAADRVPED
DG_4                MAKTNIVVVGAGFAGVYATKHLAKHYKRNKDVTITLIDRHSYFTYVTELHEIAADRVPED
DG_3                MAKTNIVVVGAGFAGVYATKHLAKHYKRNKDVTITLIDRHSYFTYVTELHEIAADRVPED
DG_2                MAKTNIVVVGAGFAGVYATKHLAKHYKRNKDVTITLIDRHSYFTYVTELHEIAADRVPED
DG_1                MAKTNIVVVGAGFAGVYATKHLAKHYKRNKDVTITLIDRHSYFTYVTELHEIAADRVPED
DG_DSM              MAKTNIVVVGAGFAGVYATKHLAKHYKRNKDVTITLIDRHSYFTYVTELHEIAADRVPED
                    ************************************************************

DG_glic             AIQYDLQRLFNRRKNVKVVTDNVTGIDRDKKEVVTENGSFPYDYVILGIGSQPNDFGTPG
DG_F                AIQYDLQRLFNRRKNVKVVTDNVTGIDRDKKEVVTENGSFPYDYVILGIGSQPNDFGTPG
DG_7                AIQYDLQRLFNRRKNVKVVTDNVTGIDRDKKEVVTENGSFPYDYVILGIGSQPNDFGTPG
DG_6                AIQYDLQRLFNRRKNVKVVTDNVTGIDRDKKEVVTENGSFPYDYVILGIGSQPNDFGTPG
DG_5                AIQYDLQRLFNRRKNVKVVTDNVTGIDRDKKEVVTENGSFPYDYVILGIGSQPNDFGTPG
DG_4                AIQYDLQRLFNRRKNVKVVTDNVTGIDRDKKEVVTENGSFPYDYVILGIGSQPNDFGTPG
DG_3                AIQYDLQRLFNRRKNVKVVTDNVTGIDRDKKEVVTENGSFPYDYVILGIGSQPNDFGTPG
DG_2                AIQYDLQRLFNRRKNVKVVTDNVTGIDRDKKEVVTENGSFPYDYVILGIGSQPNDFGTPG
DG_1                AIQYDLQRLFNRRKNVKVVTDNVTGIDRDKKEVVTENGSFPYDYVILGIGSQPNDFGTPG
DG_DSM              AIQYDLQRLFNRRKNVKVVTDNVTGIDRDKKEVVTENGSFPYDYVILGIGSQPNDFGTPG
                    ************************************************************

DG_glic             VAEHGFTLGKWEDAIRLKRHIEDVVRRGSEEHDPAKRKALLSIVIVGSGFTGTETIGELR
DG_F                VAEHGFTLGKWEDAIRLKRHIEDVVRRGSEEHDPAKRKALLSIVIVGSGFTGTETIGELR
DG_7                VAEHGFTLGKWEDAIRLKRHIEDVVRRGSEEHDPAKRKALLSIVIVGSGFTGTETIGELR
DG_6                VAEHGFTLGKWEDAIRLKRHIEDVVRRGSEEHDPAKRKALLSIVIVGSGFTGTETIGELR
DG_5                VAEHGFTLGKWEDAIRLKRHIEDVVRRGSEEHDPAKRKALLSIVIVGSGFTGTETIGELR
DG_4                VAEHGFTLGKWEDAIRLKRHIEDVVRRGSEEHDPAKRKALLSIVIVGSGFTGTETIGELR
DG_3                VAEHGFTLGKWEDAIRLKRHIEDVVRRGSEEHDPAKRKALLSIVIVGSGFTGTETIGELR
DG_2                VAEHGFTLGKWEDAIRLKRHIEDVVRRGSEEHDPAKRKALLSIVIVGSGFTGTETIGELR
DG_1                VAEHGFTLGKWEDAIRLKRHIEDVVRRGSEEHDPAKRKALLSIVIVGSGFTGTETIGELR
DG_DSM              VAEHGFTLGKWEDAIRLKRHIEDVVRRGSEEHDPAKRKALLSIVIVGSGFTGTETIGELR
                    ************************************************************

DG_glic             DWRDTLAKQYKLDPNEIQFSLMEMAPTIMNMLDRSDADKAERYMEKRGIKVMKNTGVVGV
DG_F                DWRDTLAKQYKLDPNEIQFSLMEMAPTIMNMLDRSDADKAERYMEKRGIKVMKNTGVVGV
DG_7                DWRDTLAKQYKLDPNEIQFSLMEMAPTIMNMLDRSDADKAERYMEKRGIKVMKNTGVVGV
DG_6                DWRDTLAKQYKLDPNEIQFSLMEMAPTIMNMLDRSDADKAERYMEKRGIKVMKNTGVVGV
DG_5                DWRDTLAKQYKLDPNEIQFSLMEMAPTIMNMLDRSDADKAERYMEKRGIKVMKNTGVVGV
DG_4                DWRDTLAKQYKLDPNEIQFSLMEMAPTIMNMLDRSDADKAERYMEKRGIKVMKNTGVVGV
DG_3                DWRDTLAKQYKLDPNEIQFSLMEMAPTIMNMLDRSDADKAERYMEKRGIKVMKNTGVVGV
DG_2                DWRDTLAKQYKLDPNEIQFSLMEMAPTIMNMLDRSDADKAERYMEKRGIKVMKNTGVVGV
DG_1                DWRDTLAKQYKLDPNEIQFSLMEMAPTIMNMLDRSDADKAERYMEKRGIKVMKNTGVVGV
DG_DSM              DWRDTLAKQYKLDPNEIQFSLMEMAPTIMNMLDRSDADKAERYMEKRGIKVMKNTGVVGV
                    ************************************************************

DG_glic             HEDHVDLKDGSTFPTCTLVWTAGVKATDQAKDFGLKQGRAGRILVKNNMESQDDPSIYVV
DG_F                HEDHVDLKDGSTFPTCTLVWTAGVKATDQAKDFGLKQGRAGRILVKNNMESQDDPSIYVV
DG_7                HEDHVDLKDGSTFPTCTLVWTAGVKATDQAKDFGLKQGRAGRILVKNNMESQDDPSIYVV
DG_6                HEDHVDLKDGSTFPTCTLVWTAGVKATDQAKDFGLKQGRAGRILVKNNMESQDDPSIYVV
DG_5                HEDHVDLKDGSTFPTCTLVWTAGVKATDQAKDFGLKQGRAGRILVKNNMESQDDPSIYVV
DG_4                HEDHVDLKDGSTFPTCTLVWTAGVKATDQAKDFGLKQGRAGRILVKNNMESQDDPSIYVV
DG_3                HEDHVDLKDGSTFPTCTLVWTAGVKATDQAKDFGLKQGRAGRILVKNNMESQDDPSIYVV
DG_2                HEDHVDLKDGSTFPTCTLVWTAGVKATDQAKDFGLKQGRAGRILVKNNMESQDDPSIYVV
DG_1                HEDHVDLKDGSTFPTCTLVWTAGVKATDQAKDFGLKQGRAGRILVKNNMESQDDPSIYVV
DG_DSM              HEDHVDLKDGSTFPTCTLVWTAGVKATDQAKDFGLKQGRAGRILVKNNMESQDDPSIYVV
                    ************************************************************

DG_glic             GDVSLVDQDGTGKGQPQIVQGAEATALTALTNIEAKLENKQQVEYKANYSGFMVSLGSKY
DG_F                GDVSLVDQDGTGKGQPQIVQGAEATALTALTNIEAKLENKQQVEYKANYSGFMVSLGSKY
DG_7                GDVSLVDQDGTGKGQPQIVQGAEATALTALTNIEAKLENKQQVEYKANYSGFMVSLGSKY
```

```
DG_6            GDVSLVDQDGTGKGQPQIVQGAEATALTALTNIEAKLENKQQVEYKANYSGFMVSLGSKY
DG_5            GDVSLVDQDGTGKGQPQIVQGAEATALTALTNIEAKLENKQQVEYKANYSGFMVSLGSKY
DG_4            GDVSLVDQDGTGKGQPQIVQGAEATALTALTNIEAKLENKQQVEYKANYSGFMVSLGSKY
DG_3            GDVSLVDQDGTGKGQPQIVQGAEATALTALTNIEAKLENKQQVEYKANYSGFMVSLGSKY
DG_2            GDVSLVDQDGTGKGQPQIVQGAEATALTALTNIEAKLENKQQVEYKANYSGFMVSLGSKY
DG_1            GDVSLVDQDGTGKGQPQIVQGAEATALTALTNIEAKLENKQQVEYKANYSGFMVSLGSKY
DG_DSM          GDVSLVDQDGTGKGQPQIVQGAEATALTALTNIEAKLENKQQVEYKANYSGFMVSLGSKY
                ************************************************************

DG_glic         GVANIMGWLHLSGFFAMLMKHLVNMLYFIQVYSGYYLFQYFMHEFFRTRNGRNMFRGHLS
DG_F            GVANIMGWLHLSGFFAMLMKHLVNMLYFIQVYSGYYLFQYFMHEFFRTRNGRNMFRGHLS
DG_7            GVANIMGWLHLSGFFAMLMKHLVNMLYFIQVYSGYYLFQYFMHEFFRTRNGRNMFRGHLS
DG_6            GVANIMGWLHLSGFFAMLMKHLVNMLYFIQVYSGYYLFQYFMHEFFRTRNGRNMFRGHLS
DG_5            GVANIMGWLHLSGFFAMLMKHLVNMLYFIQVYSGYYLFQYFMHEFFRTRNGRNMFRGHLS
DG_4            GVANIMGWLHLSGFFAMLMKHLVNMLYFIQVYSGYYLFQYFMHEFFRTRNGRNMFRGHLS
DG_3            GVANIMGWLHLSGFFAMLMKHLVNMLYFIQVYSGYYLFQYFMHEFFRTRNGRNMFRGHLS
DG_2            GVANIMGWLHLSGFFAMLMKHLVNMLYFIQVYSGYYLFQYFMHEFFRTRNGRNMFRGHLS
DG_1            GVANIMGWLHLSGFFAMLMKHLVNMLYFIQVYSGYYLFQYFMHEFFRTRNGRNMFRGHLS
DG_DSM          GVANIMGWLHLSGFFAMLMKHLVNMLYFIQVYSGYYLFQYFMHEFFRTRNGRNMFRGHLS
                ************************************************************

DG_glic         RQGNVLWTLPARLTLGAMWLIDCWPKIQGKESWFIDKLRLPFTWLQPAATSGASAAGADA
DG_F            RQGNVLWTLPARLTLGAMWLIDCWPKIQGKESWFIDKLRLPFTWLQPAATSGASAAGADA
DG_7            RQGNVLWTLPARLTLGAMWLIDCWPKIQGKESWFIDKLRLPFTWLQPAATSGASAAGADA
DG_6            RQGNVLWTLPARLTLGAMWLIDCWPKIQGKESWFIDKLRLPFTWLQPAATSGASAAGADA
DG_5            RQGNVLWTLPARLTLGAMWLIDCWPKIQGKESWFIDKLRLPFTWLQPAATSGASAAGADA
DG_4            RQGNVLWTLPARLTLGAMWLIDCWPKIQGKESWFIDKLRLPFTWLQPAATSGASAAGADA
DG_3            RQGNVLWTLPARLTLGAMWLIDCWPKIQGKESWFIDKLRLPFTWLQPAATSGASAAGADA
DG_2            RQGNVLWTLPARLTLGAMWLIDCWPKIQGKESWFIDKLRLPFTWLQPAATSGASAAGADA
DG_1            RQGNVLWTLPARLTLGAMWLIDCWPKIQGKESWFIDKLRLPFTWLQPAATSGASAAGADA
DG_DSM          RQGNVLWTLRARLTLGAMWLIDCWPKIQGKESWFIDKLRLPFTWLQPAATSGASAAGADA
                ********* **************************************************

DG_glic         TSAATGAAAGAAKATKTVFSLSYQYGNDPMMVFEKMPNWYYSITKALIPNQQVAFFMQKA
DG_F            TSAATGAAAGAAKATKTVFSLSYQYGNDPMMVFEKMPNWYYSITKALIPNQQVAFFMQKA
DG_7            TSAATGAAAGAAKATKTVFSLSYQYGNDPMMVFEKMPNWYYSITKALIPNQQVAFFMQKA
DG_6            TSAATGAAAGAAKATKTVFSLSYQYGNDPMMVFEKMPNWYYSITKALIPNQQVAFFMQKA
DG_5            TSAATGAAAGAAKATKTVFSLSYQYGNDPMMVFEKMPNWYYSITKALIPNQQVAFFMQKA
DG_4            TSAATGAAAGAAKATKTVFSLSYQYGNDPMMVFEKMPNWYYSITKALIPNQQVAFFMQKA
DG_3            TSAATGAAAGAAKATKTVFSLSYQYGNDPMMVFEKMPNWYYSITKALIPNQQVAFFMQKA
DG_2            TSAATGAAAGAAKATKTVFSLSYQYGNDPMMVFEKMPNWYYSITKALIPNQQVAFFMQKA
DG_1            TSAATGAAAGAAKATKTVFSLSYQYGNDPMMVFEKMPNWYYSITKALIPNQQVAFFMQKA
DG_DSM          TSAATGAAAGAAKATKTVFSLSYQYGNDPMMVFEKMPNWYYSITKALIPNQQVAFFMQKA
                ************************************************************

DG_glic         MTIMEILIGLALVAGLFTWLTSAATIAFVGVFCLSGMFYWVNIWMIPMAFACMNGSGRAF
DG_F            MTIMEILIGLALVAGLFTWLTSAATIAFVGVFCLSGMFYWVNIWMIPMAFACMNGSGRAF
DG_7            MTIMEILIGLALVAGLFTWLTSAATIAFVGVFCLSGMFYWVNIWMIPMAFACMNGSGRAF
DG_6            MTIMEILIGLALVAGLFTWLTSAATIAFVGVFCLSGMFYWVNIWMIPMAFACMNGSGRAF
DG_5            MTIMEILIGLALVAGLFTWLTSAATIAFVGVFCLSGMFYWVNIWMIPMAFACMNGSGRAF
DG_4            MTIMEILIGLALVAGLFTWLTSAATIAFVGVFCLSGMFYWVNIWMIPMAFACMNGSGRAF
DG_3            MTIMEILIGLALVAGLFTWLTSAATIAFVGVFCLSGMFYWVNIWMIPMAFACMNGSGRAF
DG_2            MTIMEILIGLALVAGLFTWLTSAATIAFVGVFCLSGMFYWVNIWMIPMAFACMNGSGRAF
DG_1            MTIMEILIGLALVAGLFTWLTSAATIAFVGVFCLSGMFYWVNIWMIPMAFACMNGSGRAF
DG_DSM          MTIMEILIGLALVAGLFTWLTSAATIAFVGVFCLSGMFYWVNIWMIPMAFACMNGSGRAF
                ************************************************************

DG_glic         GLDKWVVPYLQKVFGKWRYGTPRSLYGTDTLK
DG_F            GLDKWVVPYLQKVFGKWRYGTPRSLYGTDTLK
DG_7            GLDKWVVPYLQKVFGKWRYGTPRSLYGTDTLK
DG_6            GLDKWVVPYLQKVFGKWRYGTPRSLYGTDTLK
DG_5            GLDKWVVPYLQKVFGKWRYGTPRSLYGTDTLK
DG_4            GLDKWVVPYLQKVFGKWRYGTPRSLYGTDTLK
DG_3            GLDKWVVPYLQKVFGKWRYGTPRSLYGTDTLK
DG_2            GLDKWVVPYLQKVFGKWRYGTPRSLYGTDTLK
DG_1            GLDKWVVPYLQKVFGKWRYGTPRSLYGTDTLK
DG_DSM          GLDKWVVPYLQKVFGKWRYGTPRSLYGTDTLK
                ********************************
```

**(C)**

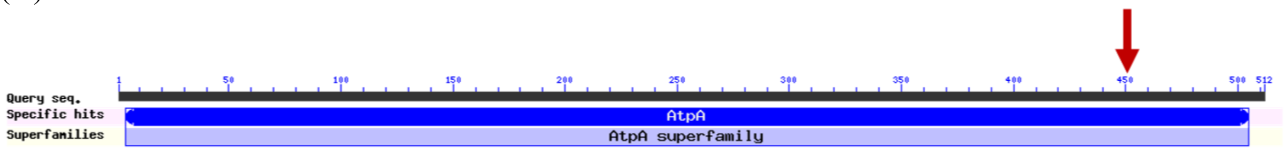


```
DG_glic          VNRMSIKTEEISSLIKKQLEGYQDDLAAEEVGTVTYIGDGIARATGLENAMANELLQFSN
DG_F             VNRMSIKTEEISSLIKKQLEGYQDDLAAEEVGTVTYIGDGIARATGLENAMANELLQFSN
DG_7             VNRMSIKTEEISSLIKKQLEGYQDDLAAEEVGTVTYIGDGIARATGLENAMANELLQFSN
DG_6             VNRMSIKTEEISSLIKKQLEGYQDDLAAEEVGTVTYIGDGIARATGLENAMANELLQFSN
DG_5             VNRMSIKTEEISSLIKKQLEGYQDDLAAEEVGTVTYIGDGIARATGLENAMANELLQFSN
DG_4             VNRMSIKTEEISSLIKKQLEGYQDDLAAEEVGTVTYIGDGIARATGLENAMANELLQFSN
DG_3             VNRMSIKTEEISSLIKKQLEGYQDDLAAEEVGTVTYIGDGIARATGLENAMANELLQFSN
DG_2             VNRMSIKTEEISSLIKKQLEGYQDDLAAEEVGTVTYIGDGIARATGLENAMANELLQFSN
DG_DSMZ          VNRMSIKTEEISSLIKKQLEGYQDDLAAEEVGTVTYIGDGIARATGLENAMANELLQFSN
DG_1             VNRMSIKTEEISSLIKKQLEGYQDDLAAEEVGTVTYIGDGIARATGLENAMANELLQFSN
                 ************************************************************

DG_glic          GSYGMALNLETNDVGIIILGDFDEIREGDQVKRTGRIMEVPVGDAMIGRVVNSLGQPVDG
DG_F             GSYGMALNLETNDVGIIILGDFDEIREGDQVKRTGRIMEVPVGDAMIGRVVNSLGQPVDG
DG_7             GSYGMALNLETNDVGIIILGDFDEIREGDQVKRTGRIMEVPVGDAMIGRVVNSLGQPVDG
DG_6             GSYGMALNLETNDVGIIILGDFDEIREGDQVKRTGRIMEVPVGDAMIGRVVNSLGQPVDG
DG_5             GSYGMALNLETNDVGIIILGDFDEIREGDQVKRTGRIMEVPVGDAMIGRVVNSLGQPVDG
DG_4             GSYGMALNLETNDVGIIILGDFDEIREGDQVKRTGRIMEVPVGDAMIGRVVNSLGQPVDG
DG_3             GSYGMALNLETNDVGIIILGDFDEIREGDQVKRTGRIMEVPVGDAMIGRVVNSLGQPVDG
DG_2             GSYGMALNLETNDVGIIILGDFDEIREGDQVKRTGRIMEVPVGDAMIGRVVNSLGQPVDG
DG_DSMZ          GSYGMALNLETNDVGIIILGDFDEIREGDQVKRTGRIMEVPVGDAMIGRVVNSLGQPVDG
DG_1             GSYGMALNLETNDVGIIILGDFDEIREGDQVKRTGRIMEVPVGDAMIGRVVNSLGQPVDG
                 ************************************************************

DG_glic          LGAIKTDKTRPIEFKAPGVMQRKSVSEPLQTGLKAIDALVPIGRGQRELIIGDRKTGKTS
DG_F             LGAIKTDKTRPIEFKAPGVMQRKSVSEPLQTGLKAIDALVPIGRGQRELIIGDRKTGKTS
DG_7             LGAIKTDKTRPIEFKAPGVMQRKSVSEPLQTGLKAIDALVPIGRGQRELIIGDRKTGKTS
DG_6             LGAIKTDKTRPIEFKAPGVMQRKSVSEPLQTGLKAIDALVPIGRGQRELIIGDRKTGKTS
DG_5             LGAIKTDKTRPIEFKAPGVMQRKSVSEPLQTGLKAIDALVPIGRGQRELIIGDRKTGKTS
DG_4             LGAIKTDKTRPIEFKAPGVMQRKSVSEPLQTGLKAIDALVPIGRGQRELIIGDRKTGKTS
DG_3             LGAIKTDKTRPIEFKAPGVMQRKSVSEPLQTGLKAIDALVPIGRGQRELIIGDRKTGKTS
DG_2             LGAIKTDKTRPIEFKAPGVMQRKSVSEPLQTGLKAIDALVPIGRGQRELIIGDRKTGKTS
DG_DSMZ          LGAIKTDKTRPIEFKAPGVMQRKSVSEPLQTGLKAIDALVPIGRGQRELIIGDRKTGKTS
DG_1             LGAIKTDKTRPIEFKAPGVMQRKSVSEPLQTGLKAIDALVPIGRGQRELIIGDRKTGKTS
                 ************************************************************

DG_glic          IAIDTILNQKDQNMICVYVAIGQKDSTVRAQVETLKKYGAMDYTIVLTAGPSEPAPMLYI
DG_F             IAIDTILNQKDQNMICVYVAIGQKDSTVRAQVETLKKYGAMDYTIVLTAGPSEPAPMLYI
DG_7             IAIDTILNQKDQNMICVYVAIGQKDSTVRAQVETLKKYGAMDYTIVLTAGPSEPAPMLYI
DG_6             IAIDTILNQKDQNMICVYVAIGQKDSTVRAQVETLKKYGAMDYTIVLTAGPSEPAPMLYI
DG_5             IAIDTILNQKDQNMICVYVAIGQKDSTVRAQVETLKKYGAMDYTIVLTAGPSEPAPMLYI
DG_4             IAIDTILNQKDQNMICVYVAIGQKDSTVRAQVETLKKYGAMDYTIVLTAGPSEPAPMLYI
DG_3             IAIDTILNQKDQNMICVYVAIGQKDSTVRAQVETLKKYGAMDYTIVLTAGPSEPAPMLYI
DG_2             IAIDTILNQKDQNMICVYVAIGQKDSTVRAQVETLKKYGAMDYTIVLTAGPSEPAPMLYI
DG_DSMZ          IAIDTILNQKDQNMICVYVAIGQKDSTVRAQVETLKKYGAMDYTIVLTAGPSEPAPMLYI
DG_1             IAIDTILNQKDQNMICVYVAIGQKDSTVRAQVETLKKYGAMDYTIVLTAGPSEPAPMLYI
                 ************************************************************

DG_glic          APYAGAAMGEEFMYNGKHVLIVYDDLSKQATSYRELSLLLRRPPGREAYPGDIFYTHSRL
DG_F             APYAGAAMGEEFMYNGKHVLIVYDDLSKQATSYRELSLLLRRPPGREAYPGDIFYTHSRL
DG_7             APYAGAAMGEEFMYNGKHVLIVYDDLSKQATSYRELSLLLRRPPGREAYPGDIFYTHSRL
DG_6             APYAGAAMGEEFMYNGKHVLIVYDDLSKQATSYRELSLLLRRPPGREAYPGDIFYTHSRL
DG_5             APYAGAAMGEEFMYNGKHVLIVYDDLSKQATSYRELSLLLRRPPGREAYPGDIFYTHSRL
DG_4             APYAGAAMGEEFMYNGKHVLIVYDDLSKQATSYRELSLLLRRPPGREAYPGDIFYTHSRL
DG_3             APYAGAAMGEEFMYNGKHVLIVYDDLSKQATSYRELSLLLRRPPGREAYPGDIFYTHSRL
DG_2             APYAGAAMGEEFMYNGKHVLIVYDDLSKQATSYRELSLLLRRPPGREAYPGDIFYTHSRL
DG_DSMZ          APYAGAAMGEEFMYNGKHVLIVYDDLSKQATSYRELSLLLRRPPGREAYPGDIFYTHSRL
DG_1             APYAGAAMGEEFMYNGKHVLIVYDDLSKQATSYRELSLLLRRPPGREAYPGDIFYTHSRL
                 ************************************************************
```

```
DG_glic         LERAAKLSDKLGGGSMTALPVIETQAGDISAYIPTNVISITDGQIFLQSDLFYAGTRPAI
DG_F            LERAAKLSDKLGGGSMTALPVIETQAGDISAYIPTNVISITDGQIFLQSDLFYAGTRPAI
DG_7            LERAAKLSDKLGGGSMTALPVIETQAGDISAYIPTNVISITDGQIFLQSDLFYAGTRPAI
DG_6            LERAAKLSDKLGGGSMTALPVIETQAGDISAYIPTNVISITDGQIFLQSDLFYAGTRPAI
DG_5            LERAAKLSDKLGGGSMTALPVIETQAGDISAYIPTNVISITDGQIFLQSDLFYAGTRPAI
DG_4            LERAAKLSDKLGGGSMTALPVIETQAGDISAYIPTNVISITDGQIFLQSDLFYAGTRPAI
DG_3            LERAAKLSDKLGGGSMTALPVIETQAGDISAYIPTNVISITDGQIFLQSDLFYAGTRPAI
DG_2            LERAAKLSDKLGGGSMTALPVIETQAGDISAYIPTNVISITDGQIFLQSDLFYAGTRPAI
DG_DSMZ         LERAAKLSDKLGGGSMTALPVIETQAGDISAYIPTNVISITDGQIFLQSDLFYAGTRPAI
DG_1            LERAAKLSDKLGGGSMTALPVIETQAGDISAYIPTNVISITDGQIFLQSDLFYAGTRPAI
                ************************************************************

DG_glic         DAGASVSRVGGDAQVKAMKKVAGTLRLDLASFRELEAFTQFGSDLDAATQAKLNRGRRTV
DG_F            DAGASVSRVGGDAQVKAMKKVAGTLRLDLASFRELEAFTQFGSDLDAATQAKLNRGRRTV
DG_7            DAGASVSRVGGDAQVKAMKKVAGTLRLDLASFRELEAFTQFGSDLDAATQAKLNRGRRTV
DG_6            DAGASVSRVGGDAQVKAMKKVAGTLRLDLASFRELEAFTQFGSDLDAATQAKLNRGRRTV
DG_5            DAGASVSRVGGDAQVKAMKKVAGTLRLDLASFRELEAFTQFGSDLDAATQAKLNRGRRTV
DG_4            DAGASVSRVGGDAQVKAMKKVAGTLRLDLASFRELEAFTQFGSDLDAATQAKLNRGRRTV
DG_3            DAGASVSRVGGDAQVKAMKKVAGTLRLDLASFRELEAFTQFGSDLDAATQAKLNRGRRTV
DG_2            DAGASVSRVGGDAQVKAMKKVAGTLRLDLASFRELEAFTQFGSDLDAATQAKLNRGRRTV
DG_DSMZ         DAGASVSRVGGDAQVKAMKKVAGTLRLDLASFRELEAFTQFGSDLDAATQAKLNRGRRTV
DG_1            DAGASVSRVGGDAQVKAMKKVAGTLRLDLASFRELEAFTQFGSDLDAATQAKLNRGRRTV
                ************************************************************

DG_glic         EVLKQPVHKPLPVEKQVIILYALTHGFLDPIPIEDITRFQDELFDFFDSNAADLLKQIRD
DG_F            EVLKQPVHKPLPVEKQVIILYALTHGFLDPIPIEDITRFQDELFDFFDSNAADLLKQIRD
DG_7            EVLKQPVHKPLPVEKQVIILYALTHGFLDPIPIEDITRFQDELFDFFDSNAADLLKQIRD
DG_6            EVLKQPVHKPLPVEKQVIILYALTHGFLDPIPIEDITRFQDELFDFFDSNAADLLKQIRD
DG_5            EVLKQPVHKPLPVEKQVIILYALTHGFLDPIPIEDITRFQDELFDFFDSNAADLLKQIRD
DG_4            EVLKQPVHKPLPVEKQVIILYALTHGFLDPIPIEDITRFQDELFDFFDSNAADLLKQIRD
DG_3            EVLKQPVHKPLPVEKQVIILYALTHGFLDPIPIEDITRFQDELFDFFDSNAADLLKQIRD
DG_2            EVLKQPVHKPLPVEKQVIILYALTHGFLDPIPIEDITRFQDELFDFFDSNAADLLKQIRD
DG_DSMZ         EVLKQPVHKPLPVEKQVIILYALTHGFLDPIPIEDITRFQDELFDFFDSNAADLLKQIRD
DG_1            EVLKQPVHKPLPVEKQVIILYALTHGFLDPVPIEDITRFQDELFDFFDSNAADLLKQIRD
                ******************************:*****************************

DG_glic         TGNLPDTDKLDAQIKAFAGGFQTSKQLAAAKD
DG_F            TGNLPDTDKLDAQIKAFAGGFQTSKQLAAAKD
DG_7            TGNLPDTDKLDAQIKAFAGGFQTSKQLAAAKD
DG_6            TGNLPDTDKLDAQIKAFAGGFQTSKQLAAAKD
DG_5            TGNLPDTDKLDAQIKAFAGGFQTSKQLAAAKD
DG_4            TGNLPDTDKLDAQIKAFAGGFQTSKQLAAAKD
DG_3            TGNLPDTDKLDAQIKAFAGGFQTSKQLAAAKD
DG_2            TGNLPDTDKLDAQIKAFAGGFQTSKQLAAAKD
DG_DSMZ         TGNLPDTDKLDAQIKAFAGGFQTSKQLAAAKD
DG_1            TGNLPDTDKLDAQIKAFAGGFQTSKQLAAAKD
                ********************************
```
